# Cesarean delivery on maternal request in Brazil: an analysis based on the perceptions of survivors of the placenta accreta spectrum

**DOI:** 10.61622/rbgo/2025rbgo79

**Published:** 2025-10-21

**Authors:** Bruna Molina Begalli, Silvana Maria Quintana, Alessandra Cristina Marcolin, Conrado Milani Coutinho, Geraldo Duarte, Marcos Masaru Okido

**Affiliations:** 1 Universidade Federal de São Carlos São Paulo SP Brazil Universidade Federal de São Carlos, São Paulo, SP, Brazil.; 2 Hospital das Clínicas Faculdade de Medicina de Ribeirão Preto Departamento de Ginecologia e Obstetrícia Ribeirão Preto SP Brazil Departamento de Ginecologia e Obstetrícia, Hospital das Clínicas, Faculdade de Medicina de Ribeirão Preto, Ribeirão Preto, SP, Brazil.

**Keywords:** Cesarean sections, Placenta accreta, Social determinants of health, Maternal mortality

## Abstract

**Objective:**

To analyze maternal request cesarean section (CDMR) through the perceptions of placenta accreta spectrum (PAS) survivors.

**Methods:**

This retrospective observational study was conducted at a university hospital, a reference center for the management of placenta accreta spectrum (PAS). Case identification was performed through a review of medical records, based on diagnoses related to placental disorders and postpartum hemorrhage. Only women who underwent puerperal hysterectomy between 2005 and 2023, with a PAS diagnosis confirmed by ultrasound conducted at the institution, were included. Eligible participants were contacted via instant messaging apps and invited to complete an online structured questionnaire, which included questions about their prior knowledge and post-event perceptions regarding cesarean delivery. Responses were compared between women in the cesarean delivery on maternal request (CDMR) group and those who underwent cesarean delivery for other indications.

**Results:**

A total of 89 cases of hysterectomy for PAS were identified during the study period. Of the 60 eligible women contacted, 41 responded to the questionnaire. Among them, 11 (26.8%) reported having requested the cesarean delivery, while 30 (73.2%) underwent the procedure for other reasons. It was found that 81.8% of women in the CDMR group had private health insurance, compared to 46.6% in the "other indications" group. High parity (≥3 pregnancies) was more common in the "other indications" group (80.0%) than in the CDMR group (36.4%). Overall, 36.6% were unaware of the risks of cesarean delivery, 48.8% did not receive adequate counseling during prenatal care, and 85.4% of participants were unaware of PAS. In the CDMR group, these values were 36.4%, 45.5%, and 72.7%, respectively. After the adverse experience, 51.2% of participants reported a change in their perception of cesarean delivery, 53.7% stated that they would have made a different choice if they had more information at the time, and 78.0% believed that the general population was unaware of the risks associated with cesarean delivery.

**Conclusion:**

The perceptions of women who experienced a "near miss" contributed to a better understanding of the process behind Brazilian women's preference for cesarean section. Decisions influenced by a pro-cesarean cultural context and misinformation tend to be poorly founded and more prone to regret. In Brazil, while CDMR is framed as an expression of autonomy, inadequate information and limited freedom can exacerbate inequalities, disproportionately affecting women in vulnerable situations.

## Introduction

The rise in cesarean delivery rates has raised global concerns regarding maternal morbidity and mortality associated with this procedure.^(1,2)^ Trend studies indicate that cesarean rates in Latin America are expected to rise from the current 42.8% to 54.3% by 2030.^(3)^ In Brazil, the average cesarean rate was 56% in 2021, with significant variation between the public system (44%) and the private health system (85%).^(4,5)^ One contributing factor to this increase is the growing prevalence of what is known as cesarean delivery on maternal request (CDMR).^(6-8)^ The preference for a specific delivery method is the result of a complex combination of multiple factors, including cultural, social, and economic influences that shape women's perceptions and expectations.^(9,10)^ Studies evaluating women's satisfaction with cesarean delivery can present biases, as most procedures are successful, leading to a predominance of favorable opinions.^(11,12)^

The increasing preference for cesarean without medical indication raises several questions. Why would a woman choose a delivery method with a higher potential for complications? Is the Brazilian pregnant woman adequately prepared to make decisions about the mode of delivery? Are they capable of critically understanding recommendations when attempting to discourage cesarean delivery? What is the woman's perception after facing a complication from an unnecessary cesarean?

Unlike most studies that have evaluated the opinions of women whose cesareans were successful; this study investigates the perceptions of women who faced serious and potentially fatal complications. It is believed that the maternal "near miss" experience may trigger survival instincts, revealing deeper perspectives that go beyond preconceived beliefs about childbirth.^(13)^

The aim of this study was to critically analyze CDMR based on the perceptions of PAS survivors. We assessed these women's level of knowledge about cesarean delivery and their perceptions of the procedure after the adverse experience. Responses from the CDMR group were compared to those of other patients. To date, no studies have evaluated this type of perception, highlighting the relevance of this research in filling gaps in the literature on the decision for cesarean delivery without medical indication. It is expected that the results will contribute to improving counseling and decision-making processes regarding delivery options, in addition to supporting the formulation of public health policies suited to our reality.

## Methods

This is an observational, retrospective and descriptive study carried out in a reference center for the treatment of patients with placenta accreta spectrum (PAS)

A search was conducted in the institution's medical records from January 2005 to January 2023. The initial screening was based on the 10th revision of the International Classification of Diseases (ICD-10), using the following codes: O72.0 – third-stage hemorrhage; O72.1 – other immediate postpartum hemorrhage; O82.2 – cesarean delivery with postpartum hysterectomy; O43.8 – other placental disorders; O43.9 – unspecified placental disorder; O44.0 – placenta previa without hemorrhage; O44.1 – placenta previa with hemorrhage; O73.0 – retained placenta without hemorrhage; and O73.1 – retained portions of placenta or membranes, without hemorrhage. Following the review of medical records, only cases of puerperal hysterectomy in women with a confirmed diagnosis of placenta accreta spectrum (PAS), based on ultrasound examination performed at the institution, were included. This selection was based on the strong association between the risk factor (prior cesarean delivery) and the acquired condition (PAS), aiming to ensure that the perceptions reported by patients were directly related to previous cesarean deliveries, considering that PAS is a complication almost exclusively observed in women with a history of cesarean section.^(14)^ Cases resulting in maternal death were excluded from the analysis. Eligible patients were individually contacted by telephone message via an instant messaging application (WhatsApp®), using the phone numbers recorded in their medical charts. The message included an explanation of the study's objectives, information about the confidentiality of the data provided, and an invitation to complete a structured online questionnaire. Up to three contact attempts were made on different days and at different times. Participation was voluntary, and no financial incentives were offered. The informed consent form was presented on the first page of the electronic questionnaire, and participants were required to provide explicit agreement to proceed.

Data were collected through a structured questionnaire, sent via instant messaging app. The questionnaire included questions regarding participants’ characteristics, as well as questions about their knowledge before the first cesarean and their opinions after the PAS experience. Additional data were obtained through the review of medical records.

The following questions were developed with categorized responses: What is your level of education? How many births have you had? How many cesareans have you had? What was your age when you had your first cesarean? What was the gestational age (length of pregnancy) at the time of your first cesarean? What was your baby's birth weight? What was your health insurance provider? What was your desire for a cesarean at the beginning of your pregnancy? What was your desire for a cesarean at the end of your pregnancy? Where was your cesarean performed? What did you know about the risks and benefits of cesarean? Did you receive counseling about the risks and benefits of cesarean? Did you know what placental accreta is? Did your opinion about cesarean change? Would you do anything different if you had the knowledge you have now? Do you know that placental accreta is a complication that occurs in women who have had cesareans? What do you think the general public knows about cesarean delivery? What is your opinion about the "cesarean law"?

To investigate the opinions of women with different preferences regarding cesarean delivery, participants were divided into two groups according to the indication for their first birth: CDMR (cesarean delivery on maternal request) for those who requested a cesarean, and "other indications" for those who believed their cesarean was not a personal choice.

The statistical analysis was conducted using SAS software version 9.4. Initially, exploratory data analysis was performed through central tendency and dispersion measures for numerical variables. Qualitative variables were summarized using absolute and relative frequencies. Associations between categorical variables were evaluated using Pearson's Chi-square test. When 25% or more of the cells in contingency tables showed expected counts less than 5, Fisher's exact test was used. A significant level of 0.05 was adopted for all analyses.

The study was approved by the Institutional Research Ethics (approval number 1.578.409), *Certificado de Apresentação de Apreciação Ética* – CAAE: 51885215.9.0000.5440.

## Results

Between 2005 and 2023, 89 cases of hysterectomy due to PAS were identified. Two maternal deaths (2.3%) occurred due to intraoperative hemorrhagic complications and were excluded from the analysis. Messages were sent to 60 women, of whom 42 responded. In 27 cases, contact information was unavailable or incorrect, and there was one refusal to participate, resulting in 41 valid responses. Eleven participants (26.8%) reported that their first cesarean delivery was on maternal request (CDMR), while 30 (73.2%) reported cesarean delivery for "other indications." The average interval between the PAS-related puerperal hysterectomy and participation in the study was 7.2 years.

Table 1 shows that 66.7% of participants had three or more pregnancies, with this proportion significantly lower in the CDMR group (36.4%) compared to the "other indications" group (80%), suggesting that women who requested cesarean delivery had, on average, lower parity. A similar pattern was observed for the number of previous cesarean deliveries: 36.4% of women in the CDMR group had three or more cesareans, compared to 70% in the other group. An association was also observed between health insurance and cesarean delivery on maternal request: 81.8% of women in the CDMR group had private health insurance, versus 46.6% in the "other indications" group.

**Table 1:** Participant characteristics

		Total n=41		CDMR (n=11, 26.8%)		Others (n=30, 73.2%)		p-value
		n(%)	Mean(SD)	n(%)	Mean(SD)	n(%)	Mean(SD)	
Age at 1st c-section (years)		41	23(6.3)	11	26(6.7)	30	22(5.9)	0.07
Education level	Elementary/High School	29(70.7)		6(54.5)		23(76.7)		0.25
	College/Postgraduate	12(29.3)		5(45.5)		7(23.3)		
Number of pregnancies	1 or 2	13(33.3)		7(63.6)		6(20.0)		0.02
	3 or more	28(66.7)		4(36.4)		24(80.0)		
Number of c-section	1 or 2	16(39.0)		7(63.4)		9 30.0)		0.07
	3 or more	25(61.0)		4(36.4)		21(70.0)		
GA at 1st c-section (weeks+days)		41	37+6 (3+2)	11	38+5(0+6)	30	37+4(3+6)	0.31
NB weight (g)		41	3005 (734)	11	3280(490)	30	2870(804)	0.11
Health insurance prenatal	Public	18(43.9)		2(18.2)		16(53.3)		0.04
	Private	23(56.1)		9(81.8)		14(46.6)		
Interval between hysterectomy and study contact (years)		41	7.2 (4.1)	11	6.6(3.6)	30	7.4(4.2)	0.55

CDMR = cesarean delivery on maternal request; GA = Gestational Age, NB = newborn. Values are presented as mean (standard deviation) for continuous variables and as number (%) for categorical variables.

As shown in table 2, participants in the CDMR group expressed a strong desire for cesarean delivery from early pregnancy, which persisted until the end of gestation. In contrast, an increase in the proportion of undecided women by the end of pregnancy was noted in the "other indications" group, suggesting that external factors may have influenced their final decision. Notably, 9.1% of women in the CDMR group still expressed a desire for vaginal birth at the end of pregnancy, while 23.3% of women in the "other indications" group reported a strong or total desire for cesarean delivery, despite stating that the surgery was not upon request—indicating possible inconsistencies between the declared indication and the actual preference. The choice of delivery facility also differed between groups: 54.5% of women in the CDMR group gave birth in private hospitals, compared to 36.7% in the other group, suggesting a correlation between access to private care and greater autonomy in choosing the mode of delivery. Regarding prior knowledge, 36.6% of all participants reported knowing little or nothing about the risks and benefits of cesarean delivery before their first surgery, with a similar proportion in the CDMR group (36.4%). Lack of adequate prenatal counseling was reported by 48.8% of participants, slightly lower in the CDMR group (45.4%). Awareness of PAS was generally low in both groups but was less frequent in the "other indications" group (90%) compared to the CDMR group (73.7%), suggesting better access to information among women who opted for cesarean delivery.

**Table 2:** Knowledge before the first c-section in women undergoing hysterectomy for placenta accreta

		Total (41)	CDMR n=11(26.8%)	Others n=30 (73.2%)	p-value
		n(%)	n(%)	n(%)	
What was your desire for a cesarean section at the beginning of your pregnancy?	None/little	24(58.6)	2(18.2)	22(73.3)	0.005
	A lot/completely	14(34.1)	8(72.8)	6(20.0)	
	Uncertain	3(7.3)	1(9.1)	2(6.7)	
What was your desire for a cesarean section at the end of your pregnancy?	None/little	21(51.2)	1(9.1)	20(66.7)	0.004
	A lot/completely	15(36.6)	8(72.7)	7(23.3)	
	Uncertain	5(12.2)	2(18.2)	3(10.0)	
What type of maternity hospital was your cesarean section performed in?	Private	17(41.5)	6(54.5)	11(36.7)	0.04
	Public	16(39.0)	1(9.1)	15(50.0)	
	Public University	8(19.5)	4(36.4)	4(13.3)	
What did you know about the risks and benefits of a cesarean section?	None/little	15(36.6)	4(36.4)	11(36.7)	0.24
	Everything /almost everything	20(48.8)	7(63.6)	13(43.3)	
	Partially	6(14.6)	0(0)	6(20.0)	
Did you receive guidance during prenatal care?	Yes	21(51.2)	6(54.5)	15(50.0)	1.00
	No	20(48.8)	5(45.5)	15(50.0)	
Did you know what placental accreta was?	Yes	6(14.6)	3 27.3)	3 (9.7)	0.31
	No	35(85.4)	8(72.7)	27(90.3)	

CDMR = cesarean delivery on maternal request

Table 3 indicates that 51.2% of women changed their opinion about cesarean delivery after experiencing the complication. This proportion was lower in the CDMR group (36.4%), indicating a possible resistance to changing opinions among those who initially desired the procedure. Similarly, 53.7% of participants stated that they would have made different decisions if they had had the knowledge they possess today—a proportion lower in the CDMR group (27.3%). Regarding perceptions of public awareness, 78% of participants believed that the general population has little or no knowledge of the issue, with similar results in the CDMR group (81.9%). Regarding the law that guarantees the right to cesarean delivery on maternal request, 63.4% of participants agreed with it, with higher agreement in the CDMR subgroup (81.9%). Notably, even after experiencing a "near miss", 31.7% of women were still unaware that PAS is a complication associated with cesarean delivery, revealing persistent information gaps despite the extreme nature of the event.

**Table 3:** Current perspectives on cesarean section in women undergoing hysterectomy for placenta accreta

		Total (41)	CDMR n=11(26.8%)	Others n=30 (73.2%)	p-value
		n(%)	n(%)	n(%)	
Has your perspective on cesarean section changed?	Not at all or little	17(41.5)	5(45.5)	12(40.0)	0.21
	Very much / completely	21(51.2)	4(36.4)	17(56.7)	
	Indifferent	3(7.3)	2(18.2)	1(3.3)	
Would you have done anything differently if you had the knowledge you have today?	Yes	22(53.7)	3(27.3)	19(63.3)	0.08
	No	15(36.6)	7(63.6)	8(26.7)	
	Vague responses	4(9.8)	1(9.1)	3(10.0)	
Do you know that a previous cesarean section can cause PAS?	Yes	28(68.3)	8(72.7)	20(66.7)	1.00
	No	13(31.7)	3(27.3)	10(33.3)	
What level of knowledge do you think laypeople have about cesarean sections?	None / little	32(78.0)	9(81.9)	23(76.7)	0.53
	Very much / totally	3(7.3)	0(0)	3(10.0)	
	Median	6(14.6)	2(18.1)	4(13.3)	
What do you think about the cesarean section law?	Agree	26(63.4)	9(81.9)	17(56.6)	0.18
	Disagree	8(19.6)	2(18.1)	6(20.0)	
	Indifferent	7(17.0)	0(0)	7(23.3)	

CDMR = cesarean delivery on maternal request

## Discussion

The results of this study reveal socioeconomic inequality between women in the CDMR group and others. There is also a lack of awareness about the risks of cesarean delivery and a likely cultural influence encouraging preference for cesarean as the method of childbirth.

In this study, women who opted for elective cesarean delivery likely belong to a higher socioeconomic group, as evidenced by greater access to private healthcare resources and family planning. This profile aligns with other national studies that highlight socioeconomic differences in birth method choice.^(7,15)^ The paradox of cesarean delivery, a procedure recognized as higher risk, being preferred by women presumed to be more informed, can be explained by the strong cultural influence in our society.^(9,16,17)^ In a study involving 455 medical students, while 96% recognized vaginal delivery as lower risk, 20.7% indicated a preference for cesarean for themselves.^(17)^

The questions designed to assess the participants’ knowledge of cesarean risks revealed significant data. Among the women in the CDMR group, 36.4% stated they knew little or nothing about the risks and benefits of the procedure. This is one of the key findings of this study, as it shows that many women request or are referred for cesarean delivery without even knowing the risks involved. This phenomenon is supported by other national studies.^(18,19)^ However, it is important to consider the potential recall bias involved in the responses. Since the accounts were collected after a critical and potentially traumatic experience, it is plausible that this event influenced how participants recall and interpret their previous desires and knowledge. This methodological limitation should be considered when analyzing reports of unawareness regarding the risks of cesarean delivery and PAS itself, as well as the changes in opinion expressed afterward.

The similar proportion of women in both the CDMR and "other indications" groups reporting that they did not receive guidance indicates a failure in prenatal care across both the public and private sectors. Zambrano et al.'s study,^(20)^ which interviewed 44 women in the private system, showed that 50% of them did not receive counseling on birth method during prenatal care. This deficiency can be explained by the cultural normalization of cesarean delivery, as well as medical convenience, where keeping the patient uninformed reduces questioning and facilitates acceptance of the procedure.^(21)^ Notably, around a third of participants, even after experiencing severe complications associated with PAS, were unaware of the association between the condition and cesarean delivery. These data underscore the complexity of health communication, which can be limited both by the quality of information provided by professionals and by the patients’ ability to fully understand it.

Table 3 presents data suggesting a high rate of regret regarding cesarean delivery. Half of the women reported that their opinion about the procedure had changed and that they would now make a different choice. This suggests a volatile decision made amidst a lack of information and a cultural pro-cesarean appeal, often resulting in doubtful decisions prone to regret. Clearly, this decision is only recognized as mistaken retrospectively in the face of an unfavorable outcome. Regret is particularly noticeable in the group of lower socioeconomic status (cesarean due to "other indications"), suggesting a complex dynamic where both patient and physician interests influence the choice of delivery method.^(21)^ This indicates that although some participants report that cesarean delivery was not an active choice, many still desired it regardless of other indications.

In this study, 73.2% of participants reported that their cesarean delivery was not by request. While it is not possible to determine with certainty, most likely, the majority desired vaginal delivery. Although most national studies show that the majority of women idealize vaginal birth for themselves,^(22,23)^ this preference can vary depending on the stage of pregnancy and the profile of the population studied.^(24)^ Preference for cesarean delivery tends to increase as birth approaches, particularly among women with private health insurance. Domingues et al.'s^(24)^ study, which analyzed 2,587 primigravidas with private insurance, showed that preference for cesarean increased from 36.1% early in pregnancy to 51.7% at the end. To illustrate that preference also depends on the studied population, Zambrano et al.'s^(20)^ study revealed that among 44 postpartum women with private health insurance, 84.1% preferred cesarean delivery. Regarding current trends, Carlotto et al.'s^(7)^ study, which interviewed 5,721 postpartum women in two hospitals in Rio Grande, Brazil, showed a 107% increase in cesarean deliveries by request from 2007 to 2016. Furthermore, the study showed that the rise in CDMR rates has been more pronounced among women with lower income and education levels.^(7)^

There is significant variation in the desire for elective cesarean delivery across countries. A 2019 review found rates ranging from 0.2% in Ireland to 24.7% in China.^(25)^ In Switzerland, the percentage of CDMR increased from 4.2% in 2002 to 10.2% in 2008.^(26)^ In Poland, 25.3% of nulliparous women desire it, but, unlike Brazil, women who prefer elective cesarean sections tend to have lower educational levels.^(27)^ Therefore, it is evident that the preference for cesarean delivery is not uniform across countries. This distinction is relevant as the pro-cesarean culture can present challenges in formulating regulations for CDMR, potentially leading to an indiscriminate increase in cesareans without medical indication.

The "cesarean law" argues for the defense of autonomy.^(28)^ Considering that autonomy is a fundamental pillar of medical ethics, there is great concern about preserving it. This principle should guarantee the patient's freedom to choose the treatment that they deem best for themselves based on their values.^(29)^ However, it is crucial to emphasize that in order to exercise autonomy within medical ethics, certain requirements must be met. The patient must understand the options presented and be fully aware of the risks and benefits after receiving precise and comprehensive counseling.^(29)^ Therefore, our study highlights that in reality, autonomy is being denied, as a significant portion of women does not receive proper counseling for decision-making.^(30,31)^

This study also reveals that the choice of Brazilian women is not entirely free, as they are immersed in a cultural context that favors cesarean delivery. Social and family pressure, along with medical persuasion, can exert indirect, subtle, and often imperceptible coercion, making it difficult for the patient to evaluate all options in a neutral and informed manner.^(30)^ The pro-cesarean cultural influence is evident, for example, in the rise of elective cesareans in recent years among women who are unaware of the risks or, even when aware, do not let this affect their decision.^(4,5)^

This and other studies reveal a concerning scenario. While cesarean delivery is still more frequent among women with higher income and education, the increase in CDMR has been more rapid among women with lower socioeconomic status and education.^(4,5)^ Therefore, it is likely that class inequality is growing, as women from higher socioeconomic backgrounds have more access to information and resources to make better decisions or reverse the negative consequences of poor choices. However, at the other extreme, there is a vulnerable group of women from lower socioeconomic backgrounds, more prone to a lack of information and strong pro-cesarean cultural pressure, who face difficulties in understanding and evaluating the complexities of this decision.^(5)^ Additionally, limited access to healthcare services exacerbates the risks, as it increases the likelihood of unintended pregnancies, repeated cesareans, placenta previa, uterine rupture, and PAS.

The main strength of this study was the sample composed exclusively of women who experienced PAS. This choice aimed to minimize the bias of overly optimistic reports typically seen in successful experiences. We believe that experiencing a life-threatening condition can have deep physical and emotional impacts that transform beliefs and behaviors, placing survival above other factors. Furthermore, the analysis of PAS, a specific complication, helped avoid confusion with other causes of hemorrhagic complications, reducing bias in interpreting the results.

The criterion used to minimize bias toward positive opinions may have generated a counteracting bias, emphasizing overly negative opinions. To mitigate this effect, we included women who had undergone hysterectomy more than a year ago to avoid the influence of recent memories. The classification between CDMR and "other indications" was based solely on the participants’ perceptions. Thus, responses may not accurately reflect preference for cesarean or vaginal birth. The "other indications" group could include cesareans performed for various reasons, ranging from legitimate medical indications to patient-requested cesareans, which may ultimately have been performed for medical convenience without the patient's full understanding.^(32)^ Additionally, some women who opted for cesarean delivery reported desiring vaginal birth, suggesting other factors influenced their decision.

## Conclusion

The perceptions of women who experienced a "near miss" contributed to a better understanding of the factors influencing Brazilian women's preference for cesarean section. Decisions shaped by a pro-cesarean cultural context and misinformation tend to be poorly grounded and more prone to regret. In Brazil, although CDMR is promoted as an expression of autonomy and free choice, it rarely offers true autonomy, as many patients lack adequate information to make informed decisions. Thus, claiming that elective cesarean section reduces social inequalities oversimplifies a complex issue. In practice, this option has expanded access to cesarean sections for women with limited access to information and resources, making them more susceptible to subsequent cesarean deliveries and, consequently, more exposed to the risks of maternal morbidity and mortality.

## Data availability

: The authors did not make the data from this article available in repositories prior to submission.
